# Trends in Self-Poisoning Suicide Attempts Before, During and After the COVID-19 Pandemic: A Seven Year Retrospective Study

**DOI:** 10.1192/j.eurpsy.2026.10673

**Published:** 2026-07-31

**Authors:** A. Žemaitis, L. Černius, M. Čistov, R. Badaras

**Affiliations:** 1 Faculty of Medicine, Vilnius University, Clinic of Psychiatry, Institute of Clinical Medicine; 2 Republican Vilnius University Hospital; 3 Vilnius University, Faculty of Medicine; 4 Faculty of Medicine, Vilnius University, Clinic of Anaesthesiology and Intensive Care, Institute of Clinical Medicine, Vilnius, Lithuania

## Abstract

**Introduction:**

The COVID-19 pandemic substantially affected mental health, increasing suicidal behaviour. Self-poisoning is a frequent suicide attempt method. Although international studies report heterogeneous findings, no retrospective analysis has been conducted in Lithuania, indicating the need for local data to clarify pandemic-related trends and support suicide prevention strategies.

**Objectives:**

The study aimed to assess the impact of the COVID-19 pandemic on the frequency of self-poisoning suicide attempts and trends across demographic groups, using data from Vilnius Republican University Hospital Toxicology Centre.

**Methods:**

The retrospective study was performed at the Toxicology Centre of Vilnius Republican University hospital and included 941 adults (≥ 18 years) hospitalised after a suicide attempt by self-poisoning from December 2017 to December 2024. Cases were grouped into pre-pandemic, pandemic (28 February 2020-29 April 2022), and post-pandemic periods, with the pandemic defined from the first confirmed case in Lithuania to the end of the national state of emergency. Variables included age, sex, place of residence (population density > 1500 and < 1500 inhabitants/. Comparisons across periods were made by the number, frequency of suicide attempts and by the distribution of these variables. Statistical analyses were performed using SPSS software (version 27.0.1.0).

**Results:**

The median age of participants was 42 years (IQR=28-56), women 70.4% (n=662), men 29.6% (n=279). A total of 324 suicide attempts by self-poisoning occurred in the pre-pandemic period, 161 during the pandemic, and 456 post-pandemic. Poisson regression showed a significant period effect (Wald (2) = 94.97, p<0.001). Compared with the pre-pandemic period, the incidence rate was lower during the pandemic (IRR=0.72, 95% CI 0.60-0.87, p=0.001), but higher post-pandemic (IRR=1.66, 95% CI 1.44-1.92, p<0.001). Gender distribution did not differ significantly between the periods (χ² =1.72, p = 0.422). In terms of residence, 573 attempts were registered in areas with >1500 inhabitants/km² and 368 in areas with <1500 inhabitants/km²; the proportion in less densely populated areas increased (32.1%, 39.1%, 44.1%), with a significant difference (χ² =11.42, p = 0.003). Median age was slightly higher in more densely populated areas; the difference was weak but significant (Spearman’s ρ=0.092, p=0.005).

**Conclusions:**

The retrospective study demonstrated that the COVID-19 pandemic influenced the dynamics of suicide attempts by self-poisoning in the Lithuanian population, with a decrease during the pandemic and a marked increase in the post-pandemic period. Gender did not influence these patterns, whereas residence was associated with differences in distribution. Findings highlight the importance of monitoring suicidal behaviour during large-scale public health crises.

**Disclosure of Interest:**

None Declared

